# Supplementary material for: Wdr47, Camsaps, and Katanin cooperate to generate ciliary central microtubules
Source: Nat Commun. 2021 Oct 4;12:5796. doi: 10.1038/s41467-021-26058-5 (PMC8490363; doi:10.1038/s41467-021-26058-5)
Supplement: Supplementary file 1 — Supplementary Information [file 41467_2021_26058_MOESM1_ESM.pdf]

Supplementary Information

Wdr47, Camsaps, and Katanin cooperate to generate ciliary central Microtubules

Hao Liu, Jianqun Zheng, Lei Zhu, Lele Xie, Yawen Chen, Yirong Zhang, Wei Zhang, Jun Zhou, Xueliang Zhu and Xiumin Yan

State Key Laboratory of Cell Biology, Shanghai Institute of Biochemistry and Cell Biology, CAS Center for Excellence in Molecular Cell Science, Chinese Academy of Sciences, 320 Yueyang Road, Shanghai 200031, China

Supplementary Figure 1, Liu et al.

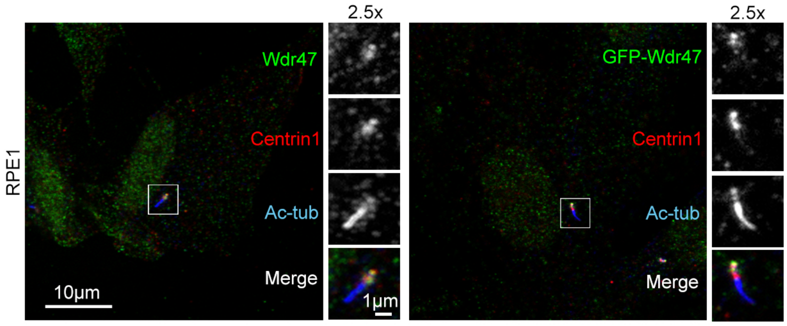

Supplementary Fig. 1. Wdr47 does not localize into primary cilia (related to Fig. 1d).
Intact or GFP-Wdr47-expressing RPE1 cells were serum-starved for 48 h to induce primary cilia and subjected to immunostaining. Centrin1 and Acetylated tubulin (Ac-tub) served as markers for the centriole and cilium, respectively. Framed regions are magnified to show details.

Supplementary Figure 2, Liu et al.

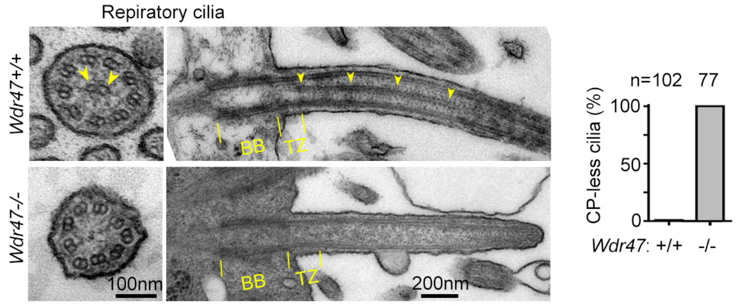

Supplementary Fig. 2. *Wdr47* deficiency abolishes the CP formation of respiratory multicilia (related to Fig. 1i).

Tracheal epithelial tissues of E18.5 mouse embryos were processed for transmission EM to visualize CP MTs (arrowheads). Experiments were performed once. BB, basal body; TZ, transition zone.

Supplementary Figure 3, Liu et al.

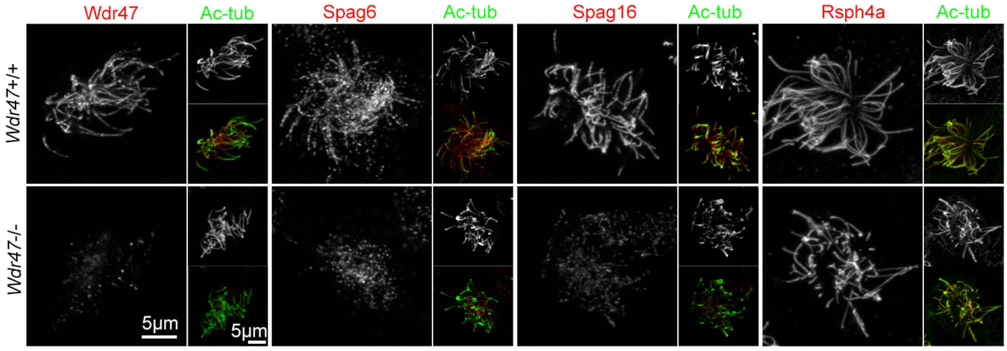

Supplementary Fig. 3. Ciliary localization of CP-associated proteins Spag6 and Spag16 markedly depends on Wdr47 (related to Fig. 2b).
mEPCs derived from *Wdr47*$^{+/+}$ or *Wdr47*$^{-/-}$ E18.5 embryos were fixed at day 10 and processed for confocal microscopy. Ac-tub marked cilia. The radial spoke subunit Rsph4a served as negative control.

Supplementary Figure 4, Liu et al.

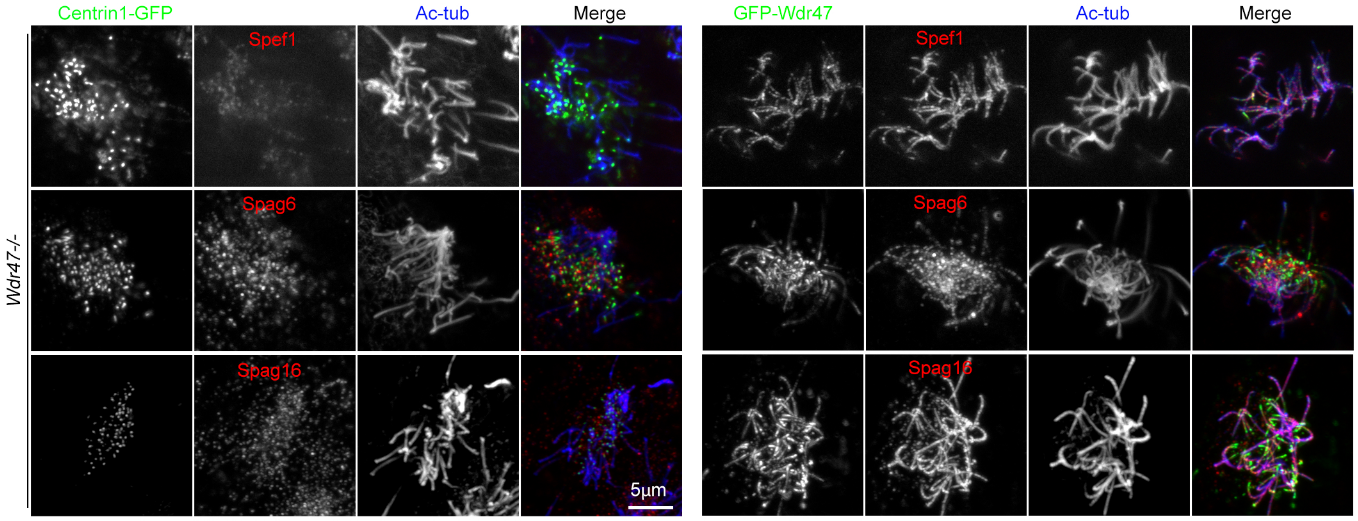

Supplementary Fig. 4. Expression of GFP-Wdr47 in Wdr47$^{-/-}$ mEPCs restores ciliary localization of CP-associated proteins Spef1, Spag6, and Spag16 (related to Fig. 2e).
mEPCs derived from Wdr47$^{-/-}$ E18.5 embryos were infected with lentivirus at one day before serum starvation (day -1) to express GFP-Wdr47 or Centrin1-GFP (negative control). The cells were fixed at day 10 and processed for confocal microscopy. Ac-tub served as cilia marker.

Supplementary Figure 5, Liu et al.

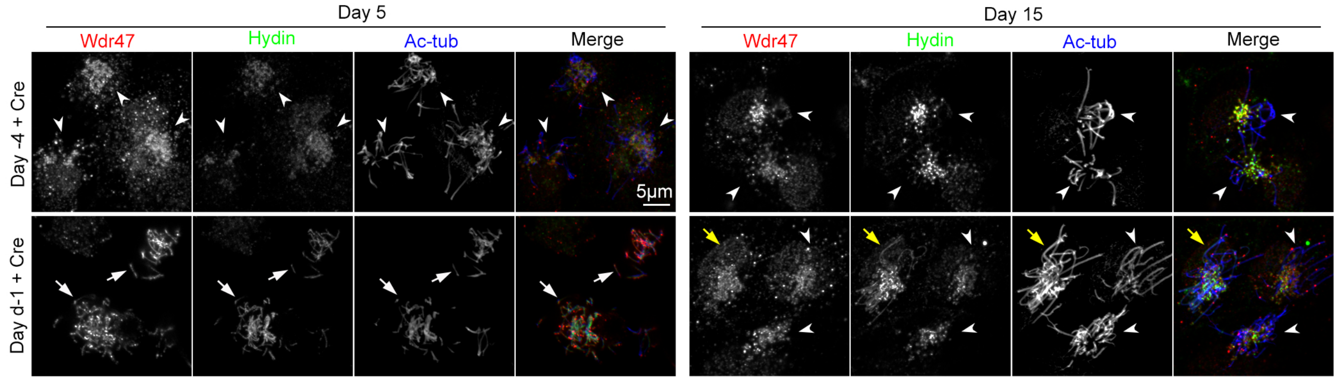

Supplementary Fig. 5. Acute depletion of Wdr47 in cultured mEPCs before or after the multiciliation induces the loss of ciliary Hydin (related to Fig. 2g-i).

Wdr47$^{flox/flox}$ mEPCs derived from P0 mice were infected with adenovirus at day -4 or day -1 to express Cre. Ciliary localizations of Wdr47 and Hydin were examined by confocal microscopy at day 5 and day 15, respectively. Ac-tub served as cilia marker. Arrowheads indicate cells with multicilia negative for both Wdr47 and Hydin, whereas arrows point to cells with double-positive multicilia. Note that the yellow arrows indicate a cell undergoing CP degeneration due to the existence of both Hydin-positive and -negative cilia.

Supplementary Figure 6, Liu et al.

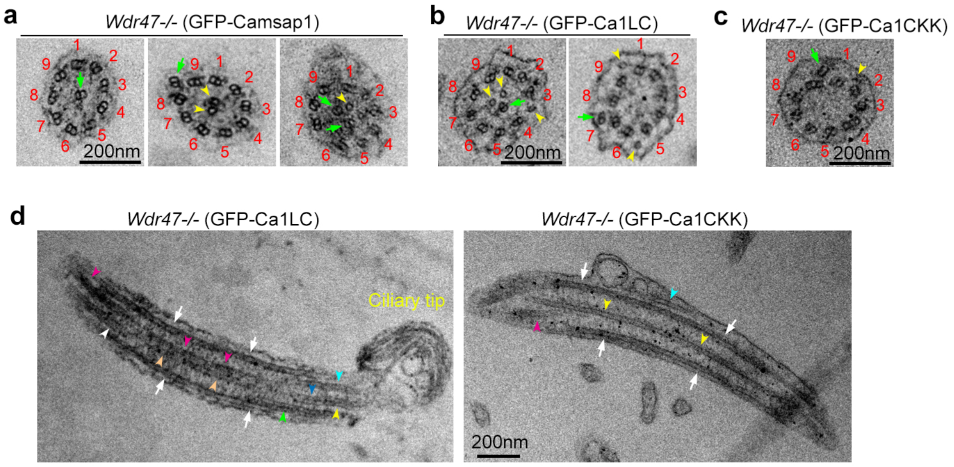

Supplementary Fig. 6. Additional examples of axonemes (related to Fig. 5f,g and Fig. 6g,h). *Wdr47* $^{-/-}$ mEPCs infected with lentivirus to express GFP-tagged Camsap1 or deletion constructs were subjected to transmission EM. Arrowheads point to central MTs. (a-c) Cross-sections showing axonemes with extra MTs that appeared as doublets (green arrows). The nine outer MT doublets are numbered randomly. (d) Longitudinal ciliary sections showing the presence of abnormal central MTs (arrowheads). Arrowheads with the same colors are used to indicate central MTs that are considered to be the same ones. White arrows indicate peripheral MT doublets.

Supplementary Figure 7, Liu et al.

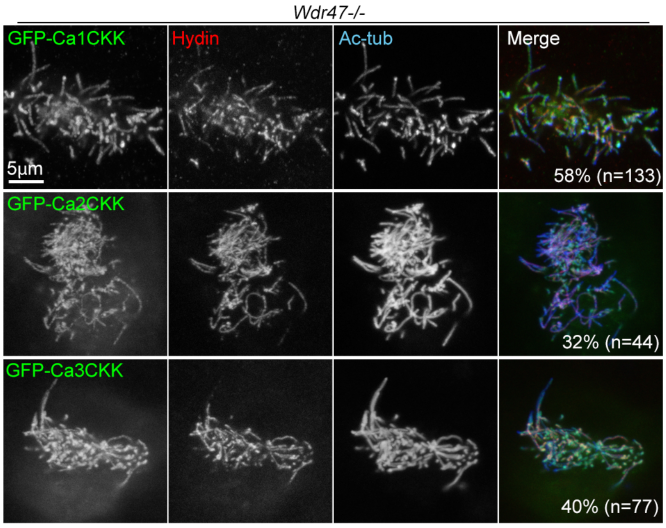

Supplementary Fig. 7. Typical confocal micrographs of *Wdr47*$^{-/-}$ mEPCs expressing the CKK constructs of Camsaps (related to Fig. 6f).
Wdr47$^{-/-}$ mEPCs were infected with lentivirus at day -1, day 2, and day 5 to overexpress GFP-tagged Camsap constructs and harvested at day 10. Hydin served as CP marker. Percentages of GFP and ciliary Hydin double-positive cells are shown.

Supplementary Table 1. List of Primers

Name	Sequence
LV-GFP-Wdr47-F	ATGACTGCTGAAGAAACGGT
LV-GFP-Wdr47-R	CTAGCCACTGTAAGTCCAGA
LV-GFP-Camsap1-F	CTCAGATCTCGAGGGATGGTGGACGCGGGC
LV-GFP-Camsap1-R	TGGACTAGTGGATCCTCACTTACGAGTCTG
LV-GFP-Camsap2-F	CTCAGATCTCGAGGGATGGGGGATGCTGCA
LV-GFP-Camsap2-R	ACTAGTGGATCCCTATGCTTTAGTGGGTAA
LV-GFP-Camsap3-F	GACTCAGATCTCGAGGGATGGTGGAAGCGGCG
LV-GFP-Camsap3-R	TGGACTAGTGGATCCCTATTTGGGGGTACCGCC
LV-GFP-WdrN-R	TTCTAACCCTGACCCCTGGC
LV-GFP-WdrC-F	AAAGAGCCTGCAAATGGAGC
LV-GFP-Ca1N1-R	TGGACTAGTGGATCCCTAGGGGGCATACAGCA
LV-GFP-Ca1N2-F	GACTCAGATCTCGAGGGCTAGTGCTGAAGCCG
LV-GFP-Ca1N2-R	TGGACTAGTGGATCCCTAACTCTGCTCCCT
LV-GFP-Ca1CC-F	CTCAGATCTCGAGGGCCAGGCAGGCACAGC
LV-GFP-Ca1CC-R	TGGACTAGTGGATCCCTAGTACTCAGCCAC
LV-GFP-Ca1CKK-F	CTCAGATCTCGAGGGACAGGTCCTAAACTC
LV-GFP-Ca2LC-F	CTCAGATCTCGAGGGACGACACAGCTGTTG
LV-GFP-Ca2CKK-F	CTCAGATCTCGAGGGACAGGACCAAAGCTT
LV-GFP-Ca3LC-F	GACTCAGATCTCGAGGGCCAACATCCACCCCC
LV-GFP-Ca3CKK-F	GACTCAGATCTCGAGGGCCCCGGCTATACAAG
LV-GFP-p60-F	GTCCGGACTCAGATCTATGACCATGAGTCTTCAAATG
LV-GFP-p60-R	CACACTGGACTAGTGCTAGCATGATCCAAACTCAAC
LV-GFP-p60^{K257A}-F	TTGGCCCACCTGGCACTGGAGCGACCCTTCTAGCTAAAG CAGT
LV-GFP-p60^{K257A}-R	ACTGCTTTAGCTAGAAGGGTCGCTCCAGTGCCAGGTGGG CCAA
LV-RFP-p60-F	CTCCGGACTCAGATCTATGACCATGAGTCTTCAAATG
LV-RFP-p60-R	CACACTGGACTAGTGCTAGCATGATCCAAACTCAAC
LV-RFP-p60^{K257A}-F	TTGGCCCACCTGGCACTGGAGCGACCCTTCTAGCTAAAG CAGT
LV-RFP-p60^{K257A}-R	ACTGCTTTAGCTAGAAGGGTCGCTCCAGTGCCAGGTGGG CCAA
Flag-Wdr47-F	GATGACAAGGGGATCCGCATGACTGCTGAAGAAACG
Flag-Wdr47-R	CTAAGCGGCCGCCTCGAGCTAGCCACTGTAAGTCCA
His-Wdr47-F	CAAATGGGTCGCGGATCCATGACTGCTGAAGAAACG
His-Wdr47-R	GTGGTGGTGGTGCTCGAGCTAGCCACTGTAAGTCCA
GST-Wdr47-F	CTGGTTCCGCGTGGATCCATGACTGCTGAAGAAACG
GST-Wdr47-R	ACGATGCGGCCGCTCGAGCTAGCCACTGTAAGTCCA
His-Camsap1 (1073-1382 aa)-F	ATGGGTCGCGGATCCGTGCCTGGCCACCGC

His-Camsap1 (1073-1382 aa)-R	GTGGTGGTGCTCGAGTCAGCTGGAGCCAGAGTG
GST-Camsap1 (1073-1382 aa)-F	GTTCCGCGTGGATCCGTGCCTGGCCACCGC
GST-Camsap1 (1073-1382 aa)-R	ATGCGGCCGCTCGAGTCAGCTGGAGCCAGAGTG
WT-F	5'-TGTCTCCCTACCCTTCATCTC-3'
WT-R	5'-CCAAATAGTCCTGCCATTAGTG-3'
KO-F	5'-CAGCCATATCACATCTGTAGAG-3'
KO-R	5'-CATGCCTTTAATCCCATCAC-3'
Cre-F	5'-CGGTCGATGCAACGAGTGATGAGG-3'
Cre-R	5'-CCAGAGACGGAAATCCATCGCTCG-3'

Supplementary Table 2 List of antibodies

Primary antibodies					
**Antigen**	**Species**	**Supplier**	**Cat. #**	**IB**	**IF**
Wdr47	rabbit	home-made		1:5000	
Wdr47	rabbit	home-made			1:500
Camsap1	rabbit	home-made		1:5000	1:500
Camsap2	rabbit	Novus	NBP1-21402	1:2000	1:200
Camsap3	rabbit	Abgent	AP18323a	1:2000	1:200
Spef1	rabbit	home-made		1:3000	1:10000
Ift80	rabbit	GeneTex	GTX109393	1:500	
Bbs3	rabbit	Proteintech Group Inc.	12676-1-AP	1:1000	
Acetylated tubulin	mouse	Sigma-Aldrich	T6793 (6-11B-1)	1:5000	1:1000
Gapdh	rabbit	Proteintech Group Inc.	10494-1-AP	1:5000	
Hydin	guinea pig	home-made			1:200
Centrin	rabbit	Proteintech Group Inc.	12794-1-AP		1:200
Spag16	rabbit	Proteintech Group Inc.	16883-1-AP	1:1000	1:200
Cep162	guinea pig	home-made			1:200
Cep290	rabbit	home-made			1:200
Flag	rabbit	Sigma-Aldrich	F7425	1:5000	
GFP	chicken	Life Technologies	A10262		1:200
GFP	rabbit	Proteintech Group Inc.	50430-2-AP	1:2000	
Ift52	rabbit	Proteintech Group Inc.	17534-1-AP	1:1000	
Spag6	rabbit	Proteintech Group Inc.	12462-1-AP		1:100
Rsph4a	rabbit	home-made			1:300
Katanin p60	rabbit	Proteintech Group Inc.	17560-1-AP	1:500	1:200

Secondary antibodies					
**Antigen**	**Species**	**Supplier**	**Cat. #**	**IB**	**IF**
anti-Mouse IgG (H+L)-HRP	goat	Life Technologies	G-21040	1:5000	
anti-Rabbit IgG (H+L)-HRP	goat	Life Technologies	G-21234	1:5000	
anti-Mouse IgG (H+L)-Pacific blue	goat	Life Technologies	P-31582		1:500
anti-Rabbit IgG (H+L)-Alexa Fluor 488	donkey	Life Technologies	A-21206		1:1000
anti-Chicken IgY-Alexa Fluor 488	goat	Life Technologies	A-11039		1:1000
anti-Mouse IgG (H+L)-Alexa Fluor 488	donkey	Life Technologies	A-21202		1:1000
anti-Guinea Pig IgG (H+L)-488	donkey	Jackson ImmunoResearch	706-545-148		1:1000
anti-Rabbit IgG (H+L)-Cy3	donkey	Jackson ImmunoResearch	711-165-152		1:1000
anti-Guinea Pig IgG (H+L)-Cy3	donkey	Jackson ImmunoResearch	706-165-148		1:1000
anti-Mouse IgG (H+L)-cy3	donkey	Jackson ImmunoResearch	715-165-151		1:1000
anti-Mouse IgG (H+L)-Alexa Fluor 647	goat	Life Technologies	A-21236		1:1000
anti-Guinea Pig IgG (H+L)-Alexa Fluor 647	donkey	Jackson ImmunoResearch	706-605-148		1:1000
