# Supplementary material for: Wdr47, Camsaps, and Katanin cooperate to generate ciliary central microtubules
Source: Nat Commun. 2021 Oct 4;12:5796. doi: 10.1038/s41467-021-26058-5 (PMC8490363; doi:10.1038/s41467-021-26058-5)
Supplement: Supplementary file 3 — Description of Additional Supplementary Files [file 41467_2021_26058_MOESM3_ESM.docx]

**Description of Additional Supplementary Files**

**Supplementary Movie 1. Rotatory beat of multicilia in *Wdr47*^-/-^ mEPCs**. mEPCs derived from E18.5 embryos of the indicated genotypes were serum starved to induce multiciliation at day 0 and cultured to day 10. Live cell imaging was performed at 140 frames per second (fps) to record ciliary motility and played back at 10 fps. Note that multicilia in *Wdr47*^+/+^ and *Wdr47*^+/-^ mEPCs beat in a back-and-forth (planar) manner. Representative frames of typical multicilia and quantification results are shown in Fig. 1g.

**Supplementary Movie 2. Planar beat of multicilia in *Wdr47*^-/-^ mEPCs expressing GFP-Wdr47.** *Wdr47^-/-^* mEPCs derived from E18.5 embryos were infected with lentivirus at one day before serum starvation (day -1) to express GFP-Wdr47 or Centrin1-GFP (negative control) and cultured to day 10. Live cell imaging was performed at 140 fps to record ciliary motility and played back at 10 fps. Note that multicilia in the cells expressing Centrin1-GFP displayed rotatory beat. Representative frames of the GFP-positive cells (arrowheads) and quantification results are shown in Fig. 2f.

**Supplementary Movie 3. Planar beat of cilia in *Wdr47*^-/-^ mEPCs overexpressing GFP-Camsaps.** *Wdr47^-/-^* mEPCs were infected with lentivirus at day -1, day 2, and day 5 to express GFP-tagged Camsaps or Centrin1 (arrowheads), respectively, and cultured to day 10. Live cell imaging was performed at 140 fps and played back at 10 fps. Note that multicilia in the cells expressing Centrin1-GFP displayed rotatory beat. Representative frames and quantification results are shown in Fig. 5b and 5c.

**Supplementary Movie 4. Planar beat of cilia in *Wdr47*^-/-^ mEPCs overexpressing GFP-CKKs.** *Wdr47^-/-^* mEPCs were infected with lentivirus at day -1, day 2 and day 5 to overexpress GFP-CKKs or Centrin1-GFP (arrowheads) and cultured to day 10. Live cell imaging was performed at 140 fps and played back at 10 fps. Note that multicilia in the cells expressing Centrin1-GFP displayed rotatory beat. Quantification results are shown in Fig. 6e.

**Supplementary Movie 5. Rotatory beat of multicilia in wild-type mEPCs expressing GFP-p60^K257A^.** mEPCs were infected with lentivirus at day -1 and day 2 to express GFP-p60, GFP-p60^K257A^, or Centrin1-GFP (arrowheads) and cultured to day 10. Live cell imaging was performed at 140 fps and played back at 10 fps. Note that multicilia in the cells expressing Centrin1-GFP displayed planar beat. Representative frames and quantification results are shown in Fig. 7e.

**Supplementary Movie 6. Rotatory beat of cilia in *Wdr47*^-/-^ mEPCs overexpressing GFP-Camsap1 and RFP-p60^K257A^.** *Wdr47^-/-^* mEPCs were infected with lentivirus at day -1, day 2, and day 5 to co-express GFP-Camsap1 with Centrin1-RFP, RFP-p60, or RFP-p60^K257A^ (arrowheads) and cultured to day 10. Live cell imaging was performed at 140 fps and played back at 10 fps. Note that cilia in the cells coexpressing GFP-Camsap1 and Centrin1-RFP or RFP-p60 mainly displayed planar beat. Representative frames and quantification results are shown in Fig. 7g.
