# Supplementary material for: Wdr47, Camsaps, and Katanin cooperate to generate ciliary central microtubules
Source: Nat Commun. 2021 Oct 4;12:5796. doi: 10.1038/s41467-021-26058-5 (PMC8490363; doi:10.1038/s41467-021-26058-5)
Supplement: Supplementary file 10 — Reporting Summary [file 41467_2021_26058_MOESM10_ESM.pdf]

nature portfolio

Corresponding author(s): Xueliang Zhu, Xiumin Yan

Last updated by author(s): 2021-8-16

Reporting Summary

Nature Portfolio wishes to improve the reproducibility of the work that we publish. This form provides structure for consistency and transparency in reporting. For further information on Nature Portfolio policies, see our Editorial Policies and the Editorial Policy Checklist.

Statistics

For all statistical analyses, confirm that the following items are present in the figure legend, table legend, main text, or Methods section.

n/a	Confirmed	
☐	☒	The exact sample size (*n*) for each experimental group/condition, given as a discrete number and unit of measurement
☐	☒	A statement on whether measurements were taken from distinct samples or whether the same sample was measured repeatedly
☐	☒	The statistical test(s) used AND whether they are one- or two-sided<br>*Only common tests should be described solely by name; describe more complex techniques in the Methods section.*
☐	☒	A description of all covariates tested
☐	☒	A description of any assumptions or corrections, such as tests of normality and adjustment for multiple comparisons
☐	☒	A full description of the statistical parameters including central tendency (e.g. means) or other basic estimates (e.g. regression coefficient) AND variation (e.g. standard deviation) or associated estimates of uncertainty (e.g. confidence intervals)
☐	☒	For null hypothesis testing, the test statistic (e.g. *F*, *t*, *r*) with confidence intervals, effect sizes, degrees of freedom and *P* value noted<br>*Give P values as exact values whenever suitable.*
☒	☐	For Bayesian analysis, information on the choice of priors and Markov chain Monte Carlo settings
☒	☐	For hierarchical and complex designs, identification of the appropriate level for tests and full reporting of outcomes
☒	☐	Estimates of effect sizes (e.g. Cohen's *d*, Pearson's *r*), indicating how they were calculated

Our web collection on statistics for biologists contains articles on many of the points above.

Software and code

Policy information about availability of computer code

Data collection	Images were collected using Delta Vision OMX SR imaging system, Leica TCS SP8 system, Olympus IX71 microscope with Andor Neo sCMOS camera , Olympus IX81 equipped with Hamamatsu EMCCD camera, FEI Quanta 250 SEM, FEI Tecnai G2 Spirit TEM, BioSpec 70/30 USR (Bruker), Olympus SZX16 Stereo Microscope, Olympus BX51 microscope.
Data analysis	3D-SIM Images were processed with SoftWoRx 1.3.0. MRI images were analyzed using ParaVision 6.0. Statistics Data were analyzed using Image J 1.53e and Graphpad Prism 8.4.0. MS/MS data were processed using MasQuant software version V1.6.10.43. Images were processed with Adobe photoshop CS6.

For manuscripts utilizing custom algorithms or software that are central to the research but not yet described in published literature, software must be made available to editors and reviewers. We strongly encourage code deposition in a community repository (e.g. GitHub). See the Nature Portfolio guidelines for submitting code & software for further information.

Data

Policy information about availability of data

All manuscripts must include a data availability statement. This statement should provide the following information, where applicable:

- Accession codes, unique identifiers, or web links for publicly available datasets
- A description of any restrictions on data availability
- For clinical datasets or third party data, please ensure that the statement adheres to our policy

Raw data of label-free quantitative proteomic results were deposited in the iProX proteome resource database (http://www.iprox.org) under accession code PXD028219. Original source data are provided as a Source Data file and the Source Data file are provided with this paper.

Field-specific reporting

Please select the one below that is the best fit for your research. If you are not sure, read the appropriate sections before making your selection.

- [x] Life sciences
- [ ] Behavioural & social sciences
- [ ] Ecological, evolutionary & environmental sciences

For a reference copy of the document with all sections, see nature.com/documents/nr-reporting-summary-flat.pdf

Life sciences study design

All studies must disclose on these points even when the disclosure is negative.

Sample size	Sample sizes were not predetermined with any statistical method and they were chosen dependent on availability and general guideline to meet requirements for statistical analyses.
Data exclusions	No data were excluded for the analyses.
Replication	Reproducibility was confirmed. The number of independent experiments is described in the figure legends.
Randomization	In our experimental design, we ensured that similar numbers of cells were randomly assigned to each group and subjected to each treatment that we tested. We imaged cells from multiple randomly-chosen fields in each experiment. For statistics, all cells in the chosen fields were scored.
Blinding	Since the cilia motility and protein localizations are different between the wild type and knockout samples, blinding is not feasible for our experiments.

Reporting for specific materials, systems and methods

We require information from authors about some types of materials, experimental systems and methods used in many studies. Here, indicate whether each material, system or method listed is relevant to your study. If you are not sure if a list item applies to your research, read the appropriate section before selecting a response.

Materials & experimental systems

n/a	Involved in the study	
[ ]	[x]	Antibodies
[ ]	[x]	Eukaryotic cell lines
[x]	[ ]	Palaeontology and archaeology
[ ]	[x]	Animals and other organisms
[x]	[ ]	Human research participants
[x]	[ ]	Clinical data
[x]	[ ]	Dual use research of concern

Methods

n/a	Involved in the study	
[x]	[ ]	ChIP-seq
[x]	[ ]	Flow cytometry
[ ]	[x]	MRI-based neuroimaging

Antibodies

Antibodies used

All antibodies used in this work were listed in supplementary table 2 with source information, catalog number and dilution information. CAMSAP2 (NBP1-21402, Novus), CAMSAP3 (AP18323a, Abgent)

Validation

All commercially available primary antibodies have been validated by the manufactures (the information of Species, Supplier and catalog number are included in Supplemental Table 2). The validation of of Rabbit anti-Wdr47, Rabbit anti-Spag16, Rabbit anti-Camsap1, Rabbit anti-Katanin p60 is based on immunofluorescence staining and immunoblotting of mouse ependymal cells. Rabbit anti-Spef1, Guinea pig anti-Hydin, Rabbit anti-Spag16, Rabbit anti-Cep162, Rabbit anti-Spag6, Rabbit anti-Rsph4a, Rabbit anti-Cep290 have been validated in previously published papers.

Rabbit anti-Spef1 home-made 10. Zheng et al., (2019). Microtubule-bundling protein Spef1 enables mammalian ciliary central apparatus formation. J Mol Cell Biol 11: 67-77.

Guinea pig anti-Hydin home-made 10. Zheng et al., (2019). Microtubule-bundling protein Spef1 enables mammalian ciliary central apparatus formation. J Mol Cell Biol 11: 67-77.

Rabbit anti-Camsap2 NBP1-21402 (Novus) Jiang, K. et al. (2014). Microtubule minus-end stabilization by polymerization-driven CAMSAP deposition. Dev Cell 28, 295-309.

Rabbit anti-Camsap3 AP18323a (Abgent) Jiang, K. et al. (2014). Microtubule minus-end stabilization by polymerization-driven CAMSAP deposition. Dev Cell 28, 295-309 (2014).

Rabbit anti-Cep162 home-made 8. Zhao et al. (2019). Parental centrioles are dispensable for deuterosome formation and function. EMBO Rep 20: e46735.

Rabbit anti-Spag6 12462-1-AP (Proteintech) Zheng et al., (2019). Microtubule-bundling protein Spef1 enables mammalian ciliary central apparatus formation. J Mol Cell Biol 11: 67-77.

Rabbit anti-Rsph4a home-made Zheng et al., (2021). Distinct architecture and composition of mouse axonemal radial spoke head

revealed by cryo-EM. Proc Natl Acad Sci 118 (4): e2021180118.
Rabbit anti-Cep290 homemade 4. Duan et al. (2021). Rabl2 GTP hydrolysis licenses BBSome-mediated export to fine-tune ciliary signaling. EMBO J 40:e105499.

Eukaryotic cell lines

Policy information about cell lines

Cell line source(s)	HEK293T (ATCC), RPE1(ATCC)
Authentication	The cell lines have been authenticated by STR profiling by the vendor. No further authentication was performed.
Mycoplasma contamination	Mycoplasma contamination was tested and found negative.
Commonly misidentified lines (See ICLAC register)	No commonly misidentified cell lines were used.

Animals and other organisms

Policy information about studies involving animals; ARRIVE guidelines recommended for reporting animal research

Laboratory animals	Wild-type P0 and 4-week male or female C57BL/6J mice were used for primary cell culture. 2-month Wdr47Kof/+ mice were crossed with 2-month FVB EIIa-Cre mice to generate Wdr47+/- mice, which were used to produce P0 Wdr47-/- mice. Wdr47+/- mice were self-crossed more than 10 generations to obtain the Wdr47+/- mice with C57BL/6J background. Wdr47Kof/+ mice were crossed with Wdr47+/- mice to generate P14 Wdr47Kof/- mice. Wdr47Kof/+ mice were crossed with Flp mice to produce Wdr47flox/+ mice, which were further crossed with GFAP-Cre mice to produce P10 or P21 Wdr47flox/flox ;GFAP-Cre mice. The sex of the mice used in this study was not recorded before experiments. The mice were housed under specific-pathogen-free (SPF) conditions in cages and a 12/12-hr light/dark photoperiod at 20-26 °C. The humidity of the housing room was maintained at 40-70% humidity.
Wild animals	No wild animals were used.
Field-collected samples	No field-collected samples were used.
Ethics oversight	Experiments involving mouse tissues were performed in accordance with protocols approved by the Institutional Animal Care and Use Committee of CAS Center for Excellence in Molecular Cell Science, Institute of Biochemistry and Cell Biology, Chinese academy of Sciences.

Note that full information on the approval of the study protocol must also be provided in the manuscript.

Magnetic resonance imaging

Experimental design

Design type	MRI experiments were performed to confirm hydrocephalus in mice.
Design specifications	MRI Brain images were obtained in the Axial view.
Behavioral performance measures	No behavioral performance measures were performed.

Acquisition

Imaging type(s)	Longitudinal magnetic resonance imaging
Field strength	7
Sequence & imaging parameters	The imaging parameters were: Acquisituon Time: 06:24. Protocol Name: T1_RARE/ Bruker: RARE. Resolution matrix: 256x256 (a). Number of Slices: 55 unary, no units. Fov : 37.81 mm x 31.71 mm. Repetition time (TR): 2777.2 ms. Echo time (TE): 34 ms. Filp angle: 90 degree.
Area of acquisition	MRI brain obtained in Axial view,<br>No. of Slices: 55 unary, no units, Slice thickness (0.5 mm)

Diffusion MRI ☐ Used ☒ Not used

Preprocessing

Preprocessing software	ParaVision 6.0
Normalization	Not relevant (we did not perform functional magnetic resonance imaging analyses).
Normalization template	Not relevant (we did not perform functional magnetic resonance imaging analyses).

Noise and artifact removal	Not relevant (we did not perform functional magnetic resonance imaging analyses).
Volume censoring	Not relevant (we did not perform functional magnetic resonance imaging analyses).

Statistical modeling & inference

Model type and settings	Not relevant. We did not perform functional magnetic resonance imaging analyses and no statistical modeling was performed.
Effect(s) tested	Not relevant. We did not perform functional magnetic resonance imaging analyses.

Specify type of analysis: ☐ Whole brain ☐ ROI-based ☐ Both

Statistic type for inference (See Eklund et al. 2016)	Not relevant (we did not perform functional magnetic resonance imaging analyses)
Correction	*Describe the type of correction and how it is obtained for multiple comparisons (e.g. FWE, FDR, permutation or Monte Carlo).*

Models & analysis

n/a	Involved in the study
☒	☐ Functional and/or effective connectivity
☒	☐ Graph analysis
☒	☐ Multivariate modeling or predictive analysis
